# Persistent Left-Sided Superior Vena Cava: A Case Report

**DOI:** 10.7759/cureus.67935

**Published:** 2024-08-27

**Authors:** Matthew Hentges, Karson Schroeder, Aleesha Somani

**Affiliations:** 1 General Surgery, Kansas City University, Kansas City, USA

**Keywords:** persistent left-sided superior vena cava (plsvc), anatomy and physiology, ultrasound-guided, general and vascular surgery, central line insertion

## Abstract

A persistent left-sided superior vena cava (PLSVC) is the most common thoracic venous anomaly. However, it is still quite rare in the general population. PLSVC occurs during the embryological stages and is seen mostly in patients with congenital heart disease. Normally during development, the left anterior cardinal vein will regress and obliterate to form the ligament of Marshall. In cases of PLSVC, the left anterior cardinal vein persists and can become a persistent left superior vena cava (SVC). There are different anatomical variants of a left-sided SVC, most commonly presenting with both a right and a left SVC. In some PLSVC cases, there is an isolated left SVC. Though rare, this anomaly is not without clinical significance. This case report describes a 48-year-old male with incidental findings of isolated PLSVC seen on chest X-ray after the placement of a temporary dialysis catheter. This report will also describe the incidence/prevalence, embryological origin including anatomical variants, and clinical implications of PLSVC.

## Introduction

This case report presents an incidental finding of persistent left-sided superior vena cava (PLSVC). This is an anomaly that forms during the embryological development of the heart and is the most common congenital venous anomaly [1]. A review of the current research suggests that the incidence of PLSVC is 0.2-3% in the general population. However, this number increases to 1.3-11% in populations with congenital heart disease [1]. While this anomaly is often discovered incidentally since it is asymptomatic, it can have vast clinical implications, especially during cardiac procedures. This report will discuss the incidence, embryological development, and clinical implications of a PLSVC. 

## Case presentation

**Figure 1:**
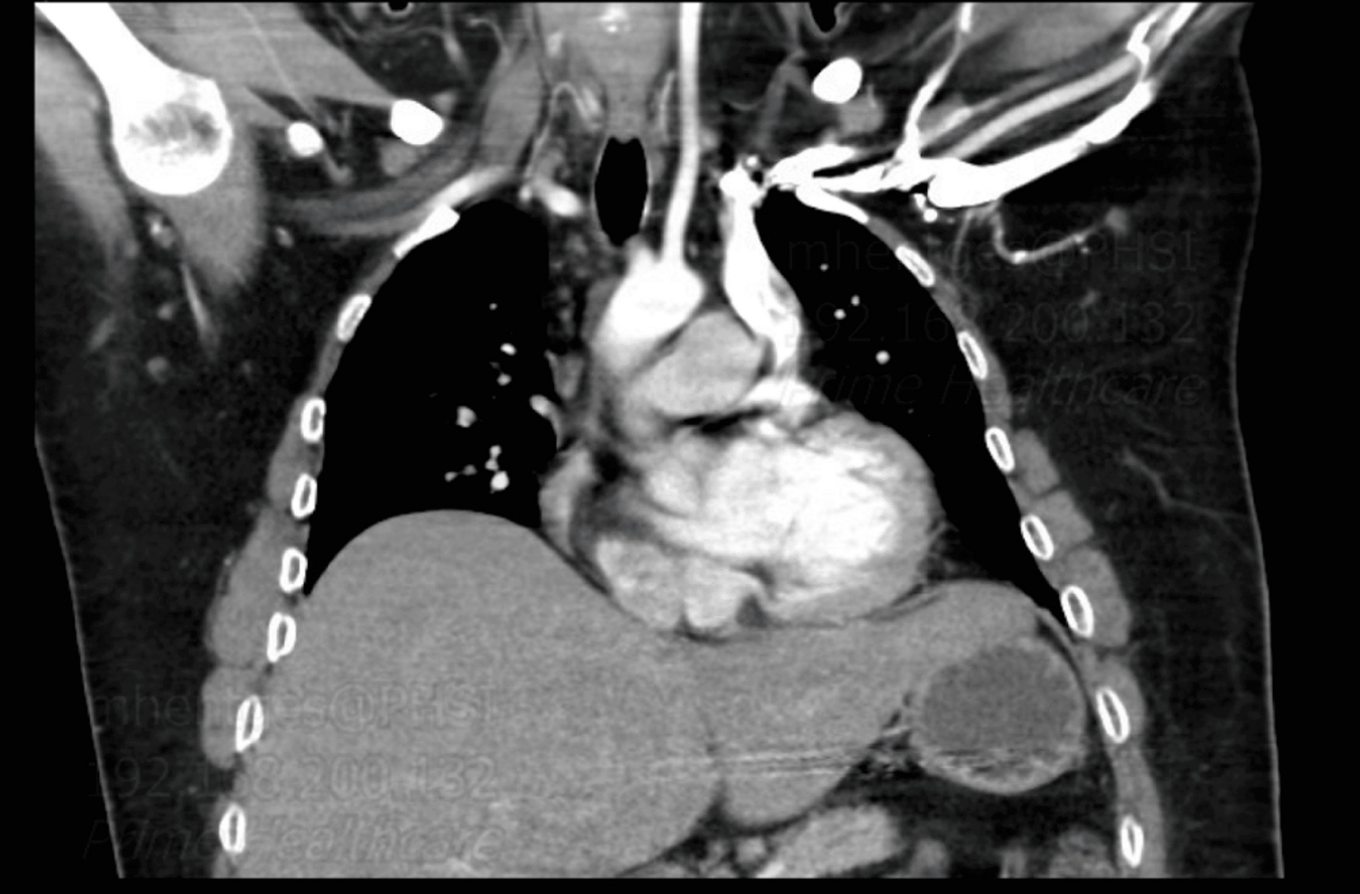
CT of the chest, 2020. Left-sided SVC dumping into the coronary sinus was noted. The aortic arch with the left common carotid can also be visualized. SVC: superior vena cava

**Figure 2:**
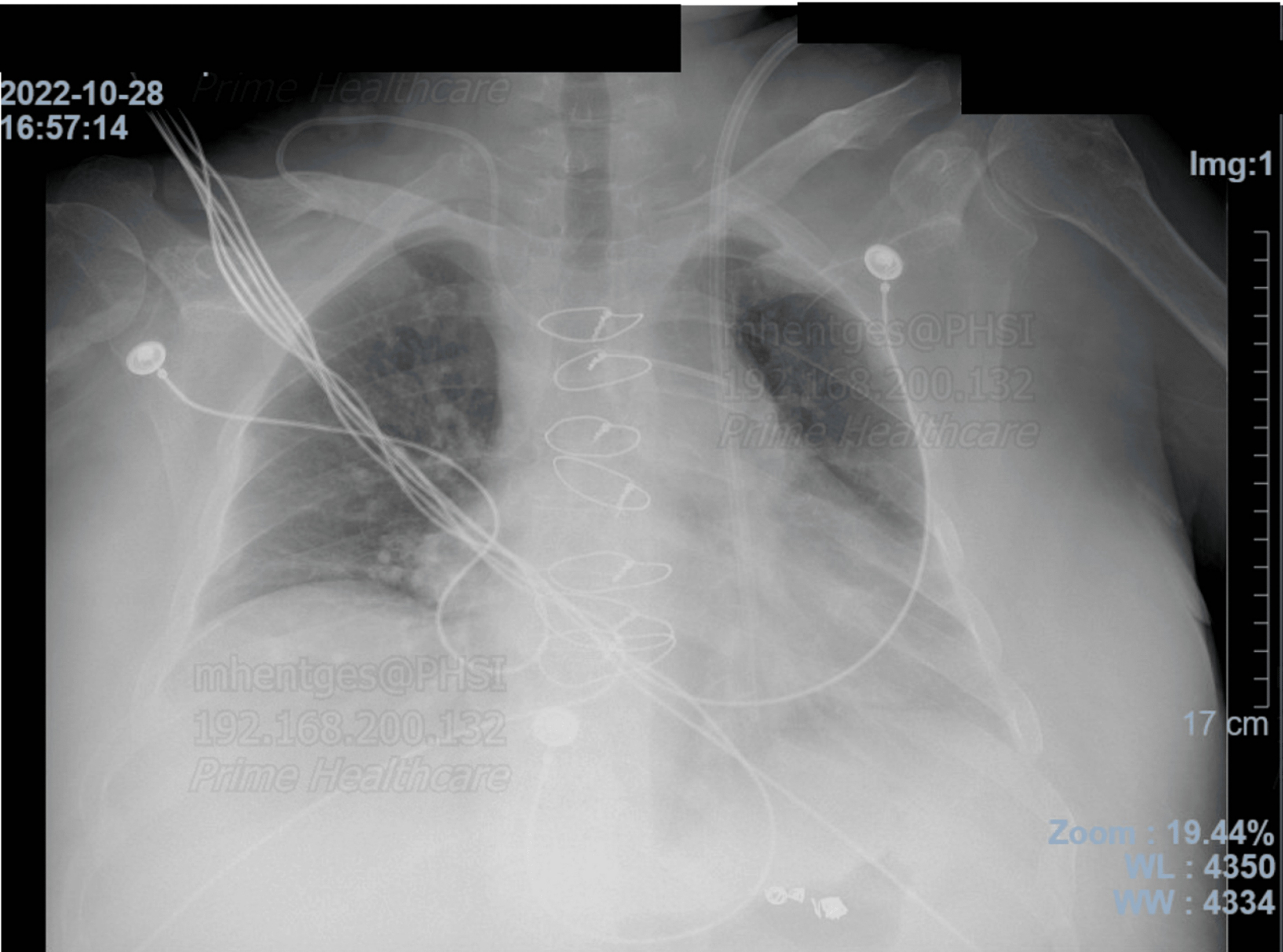
Chest X-ray, 2022. The temporary dialysis catheter was visualized descending down the left chest via a left-sided SVC dumping into the coronary sinus. SVC: superior vena cava

This is the case of a 48-year-old male with a history of coronary artery disease s/p triple vessel bypass in 2020, degenerative disc disease, depression/anxiety, gastroesophageal reflux disease (GERD), diabetes mellitus (DM), gastroparesis, seizures, hyperlipidemia, and insomnia presenting in October 2022 to general surgery for the placement of a temporary dialysis catheter. He presented to the emergency department (ED) one day prior with tachycardia and hypotension. Previously, he was admitted in June 2022 for cardiac arrest, septic shock, acute respiratory failure requiring intubation, acute renal failure requiring temporary hemodialysis, and DM with foot ulcer. He was eventually discharged to an acute rehab facility and then discharged home with outpatient follow-up with nephrology and cardiology. There was little mention of a left-sided superior vena cava (SVC) at that time. Upon subsequent presentation to the ED in October 2022, he was complaining of weakness and fatigue for 1-2 days. He was found to be in acute renal failure and in need of a temporary dialysis catheter to start hemodialysis. The surgery resident on call placed a left-sided internal jugular temporary dialysis line, and a chest X-ray was obtained to verify the placement (Figure 1 and Figure 2). Upon review of the chest X-ray, the line was visualized descending down the left chest without expected crossing over. At that time, the patient's old imaging records were reviewed, and the patient's anatomy was confirmed to have an isolated left-sided SVC. This was confirmed via a CT of the chest dating back to 2020 but was never mentioned in his cardiac history or prior notes. The patient went on to receive hemodialysis over the following week with improvement and discharge to skilled nursing on November 4, 2022, with outpatient hemodialysis and follow-up with cardiology and nephrology.

## Discussion

The PLSVC is most often an incidental finding. It can usually be seen during cardiac imaging, for example, during an X-ray post-central line placement. Other cases also describe discovery during cardiac procedures such as placement of a permanent pacemaker. A dilated coronary sinus on echo should indicate a high clinical suspicion for a left-sided SVC [2]. 

In the normal anatomy of the venous return from the superior portion of the body, the right subclavian vein merges with the right internal and external jugular vein to form the right brachiocephalic vein. The left subclavian vein merges with the left internal and external jugular vein to form the left brachiocephalic vein. The left brachiocephalic vein traverses across the body to meet with the right brachiocephalic vein and form the SVC. 

**Figure 3:**
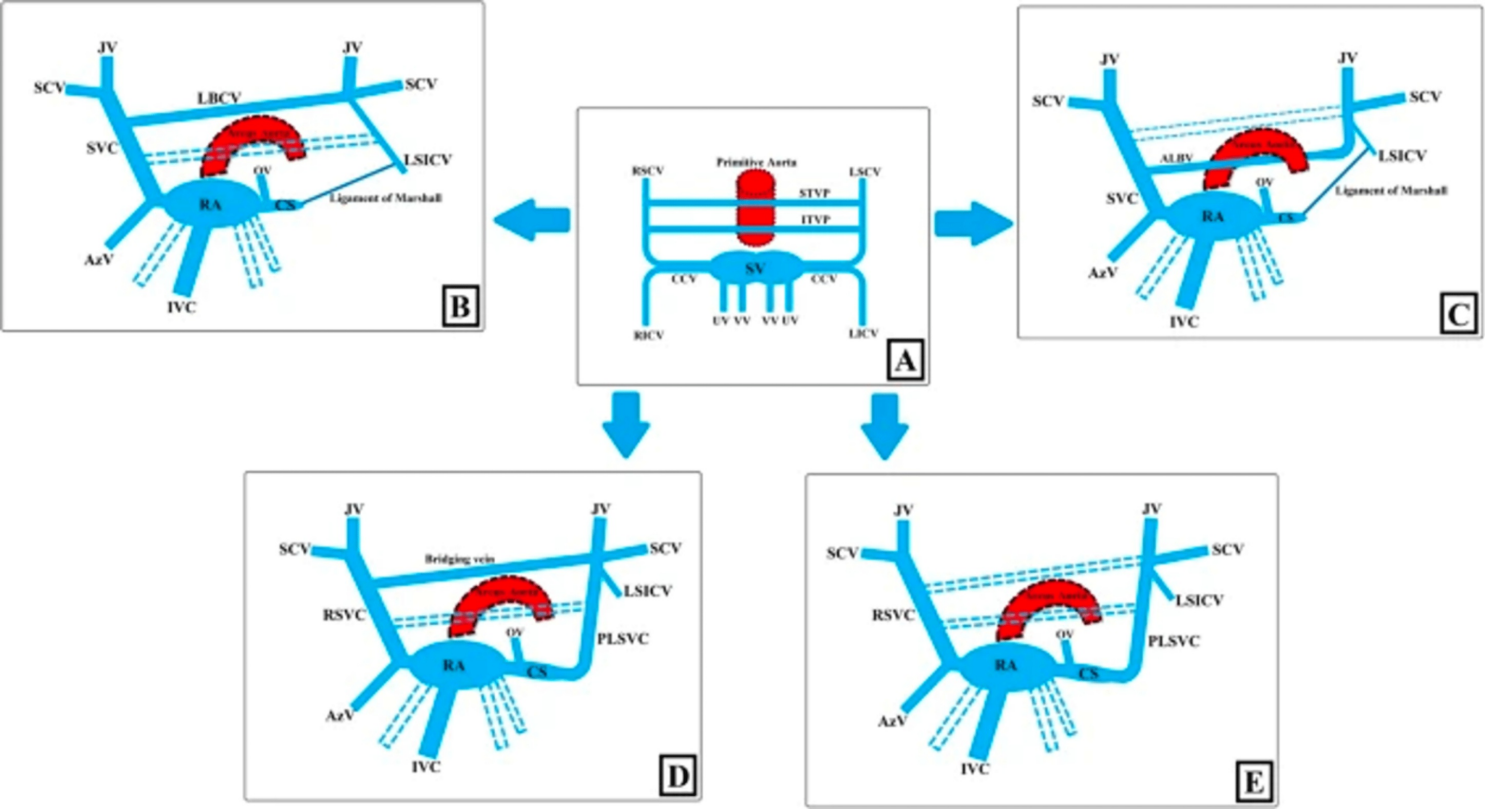
Venous return developmental stages and embryological variations of PLSVC. PLSVC: persistent left-sided superior vena cava

During development, the left anterior cardinal vein regresses and becomes the ligament of Marshall. If the left anterior cardinal vein persists, it becomes a PLSVC. There are different anatomical variants that can form during the development of PLSVC; see Figure 3. Around 80-90% of cases also show the presence of a right-sided SVC. Around 30% of those have a left brachiocephalic vein also present which forms a connection between the left-sided SVC and the right-sided SVC. Around 10% of cases show an isolated SVC present. In almost 90% of cases, the left SVC drains into the coronary sinus which then drains into the right atrium. However, in around 10% of cases, the left-sided SVC can drain directly into the left atrium, creating a right-to-left shunt [1].

Once diagnosed, it may be prudent to rule out other cardiac defects (such as atrial septal defect (ASD)). If a person is found to have a PLSVC, then a cardiac workup or review of prior imaging to look for additional defects should be initiated. The most common congenital cardiac anomaly associated with a PLSVC is a sinus venosus defect (SVD), a type of ASD. SVD accounts for 4-11% of all ASDs. A multidetector CT scan can be utilized to easily see if any of these defects are present [3]. It is necessary to check for additional defects to prevent hemodynamic complications during or after treatment. 

The presence of a PLSVC can complicate various cardiac procedures including Swan-Ganz catheter, permanent implantable cardioverter-defibrillator (ICD), or pacemaker placement. When unaware of the PLSVC or with improper technique, cardiac procedures may lead to cardiac tamponade, arrhythmias, cardiogenic shock, or coronary sinus thrombosis [4]. The presence of a PLSVC is a relative contraindication for retrograde cardioplegia administration [5]. In retrograde cardioplegia, a catheter is placed in the coronary sinus, and flow is administered retrograde. In a patient with a PLSVC, the infusion can travel up the PLSVC unless adequately clamped. Even with appropriate clamping of a PLSVC, accessory veins may exist that lead to a steal of cardioplegia solution leading to inadequate myocardial protection [6].

In cases where the PLSVC drains into the left atrium, most are asymptomatic due to the small right-to-left shunt this creates. In patients where the shunt is more pronounced, this can lead to severe cyanosis, reduced exercise tolerance, syncope, and progressive fatigue. PLSVC is also a common cause of atrial fibrillation (AFib). In patients with PLSVC and AFib, the anomalous vein can be ablated to prevent the recurrence of AFib [7]. Although a PLSVC can slightly complicate access to the right heart, it is not an absolute contraindication. Right atrial and right ventricular leads for implantable pacemakers have been successfully placed through a PLSVC. As catheters and techniques improve over time, accessing the right heart through a PLSVC will become less of a risk to the patient. In most patients, a PLSVC will be identified incidentally on imaging or during a procedure [8]. It is important once found to check for other cardiac and vascular anomalies that may also be present. If the patient is asymptomatic, simply monitoring over time and documentation of findings is adequate management. 

## Conclusions

Although the incidence of patients with a persistent left SVC is minimal, recognition of the anomaly and a cardiac workup to follow are important for all physicians. If unknown, a PLSVC can complicate procedures like central line placement or dialysis line placement. A PLSVC may lead to the physician thinking that the line has been placed into the carotid and down into the aorta on a confirmatory chest X-ray. If found during a procedure or on imaging, further cardiac and vascular evaluation to look for other anomalies should take place. It is important to note that PLSVC does not preclude the insertion of catheters. Furthermore, it is also important to recognize that the vast majority of these patients will be asymptomatic and live the rest of their lives with no complications. Regardless, PLSVC is not without risk. Hence, this should be discussed with the patient, and alternative access sites may need to be considered.
